# Soccer (Football Association) and chronic traumatic encephalopathy: A short review and recommendation

**DOI:** 10.1590/1980-57642016dn11-030002

**Published:** 2017

**Authors:** Ricardo Nitrini

**Affiliations:** 1 MD, PhD. Chefe do Departamento de Neurologia e da Divisão de Clínica Neurológica do Hospital das Clínicas da Faculdade de Medicina da Universidade de São Paulo, SP, Brazil.

**Keywords:** chronic traumatic encephalopathy, soccer, football, heading, head trauma, encefalopatia traumatica crônica, futebol, futebol de associação, cabeceio, trauma de crânio

## Abstract

Chronic traumatic encephalopathy (CTE) was initially described in boxers, but in recent years it has been reported in other settings, particularly in contact sports and military personnel. Soccer (football association) had previously been (and still is) considered relatively safe when compared to other sports, such as American football. However, a few cases of professional soccer players with CTE have been reported in the last few years. It is still unknown how frequent this condition is in soccer players, and the role played by heading the ball remains elusive. Other traumas to the head, face and neck caused by contact with another player's head, arm or other body parts are among the most frequent in soccer. In spite of the lack of more in-depth knowledge, there is reasonable evidence for recommending severe punishment (red card and suspension for several matches) for players causing avoidable trauma to another player's head.

## RESUMO

Encefalopatia traumática crônica (ETC) foi inicialmente descrita em boxeadores, mas nos últimos anos tem sido relatada em outras situações, particularmente nos esportes de contato e no pessoal militar. O futebol ou futebol de associação foi (e ainda é) considerado relativamente mais seguro quando comparado a outros esportes, como o futebol americano. No entanto, alguns casos de jogadores de futebol profissional com CTE foram relatados nos últimos anos. Ainda não se sabe a frequência dessa condição nos jogadores de futebol bem como qual o papel desempenhado por cabecear a bola. Outros traumas de crânio e pescoço causados pelo contato com o crânio, braço ou outras partes do corpo de outros jogadores estão entre os mais frequentes no futebol. Apesar da falta de um conhecimento mais aprofundado, há razoável evidência para recomendar punição severa (cartão vermelho e suspensão por várias partidas) para jogadores que causarem trauma evitável na cabeça de outro jogador.

## INTRODUCTION

In the last edition of the Congress Brain, Behavior and Emotions, held in Porto Alegre, Brazil, in June 2017, I delivered a plenary lecture as co-chair of the event. I chose to speak on the relationship between soccer (or football association) and chronic traumatic encephalopathy (CTE), because our group reported one of the first cases of CTE in a professional soccer player,^1^^,^ 2 and given that it is a relatively recent topic which deserves reflection, especially on prevention procedures.

## PERSONAL EXPERIENCE

My story began when watching a television documentary about a lawsuit filed by former football players against the USA National Football League, the NFL, involving millions of dollars. The process, which later came to be well known due to the motion picture *Concussion*, began when a Nigerian forensic pathologist working in USA, Bennett Omalu, published a case report of a former professional football player with CTE in Neurosurgery.^3^ Clinical manifestations included severe behavioral changes and cognitive decline, somewhat similar to a frontotemporal dementia. There were attempts to misrepresent Omalu and to stifle results, but with new cases from other researchers, most notably by the neuropathologist Ann McKee,^4^ it became evident that CTE had occurred in several other former players and was not a rare condition.

CTE was first described in boxers under the name "punch-drunk syndrome" (Martland, 1928),^5^ later renamed "dementia pugilistica" (Millspaugh 1937),^6^ and finally as Chronic Traumatic Encephalopathy (Critchley 1957).^7^ The neuropathological characterization of CTE was well described by Corsellis et al. (1973).^8^ Our group had previous experience with CTE because a few years earlier we saw a former boxer who presented with clinical features of Alzheimer's disease, but whose post-mortem neuropathological examination confirmed CTE.^9^

When watching the documentary, I immediately remembered that a soccer player with a presumed diagnosis of Alzheimer's disease was registered in our outpatient clinic at the Hospital das Clínicas of the University of São Paulo Medical School: Bellini, the great captain of the Brazilian team that won the first the Football World Cup for Brazil, in 1958. Bellini was being treated for advanced dementia and for this reason I immediately sought contact with his family.

In a long conversation with Mrs. Giselda Bellini, Bellini's wife, I explained to her my hypothesis that he might have CTE rather than Alzheimer's disease. This interview, and the facts that followed, were reported by Mrs. Bellini in her book entitled "Bellini: the first captain champion",^10^ and so I feel entitled to report them. She listened to me carefully and confessed to me that she had long been intrigued by the number of teammates of Bellini who, according to her, had had Alzheimer's disease or some form of dementia. She talked to his children about my hypothesis and there was agreement for brain donation for neuropathological examination upon his death, which occurred shortly after.

The neuropathological examination performed at the facilities of the Department of Pathology of the Hospital das Clínicas of the University of São Paulo Medical School by the neuropathologist Lea T. Grinberg confirmed the presence of macroscopic and microscopic alterations of CTE. There were also other changes revealing a diagnosis of mixed dementia based on the presence of lacunar infarcts, hippocampal sclerosis, moderate stage Alzheimer's disease and TDP43 protein deposits.^1^^,^ 2

Shortly before the XVIII International Congress of Neuropathology, held in Rio de Janeiro in September 2014, the neuropathologist Ann McKee had the opportunity to see the slides from the neuropathological examination and confirmed the diagnosis of CTE. The case was presented at the congress and was probably the first record (as an abstract) of dementia caused by CTE in a soccer player.^1^

The impact of the report was great both within the country^11^^,^ 12 and abroad.^13^^,^ 14

## OTHER STUDIES ON CTE IN SOCCER PLAYERS

Previous clinical studies had observed poor performance of former soccer players on neuropsychological tests,^15^^-^ 17 but without neuropathological examination. In June 2014, the BBC reported a case in which the review of the neuropathological study performed several years earlier confirmed the diagnosis of CTE. 18

A few months later, in the same year, Hales and colleagues published the case report of a retired professional soccer player who died from dementia in Neurology as a full paper. The neuropathological examination, revealed the characteristics of CTE associated with diffuse plaques, some neuritic plaques and cytoplasmic inclusions of TDP43, thus a mixed dementia.^19^

The full paper of the Bellini case was published in 2016.^10^ In the beginning of 2017, only two other cases of dementia with neuropathological demonstration of CTE, besides Bellini's case, had been identified. A case with neuropathological demonstration of amyotrophic lateral sclerosis in a soccer player had also been described.^20^

In 2017, Ling et al. presented a study in the UK in which 12 retired football players with dementia were followed for a long period to death, 6 of whom underwent neuropathological examination. Of these, four had CTE which was also associated with other neuropathological changes consistent with the diagnosis of mixed dementia.^21^

## WHAT WE KNOW AND WHAT WE NEED TO KNOW

Do these six cases with pathologically-confirmed diagnoses allow us to know whether CTE is really caused by soccer? And if so, how frequent is it?

It is likely that CTE is caused by soccer, but we do not know how frequent it is and cannot evaluate the risks. More studies are needed and some are currently underway in several centers, including by our group led by Renato Anghinah and Claudia Costa Leite at the Hospital das Clínicas of the University of São Paulo Medical School.

Other issues that warrant further research are related to the clinical diagnosis of CTE and its treatment, particularly how to avoid evolution from the sparse deposits of phosphorylated tau proteins in the brain to full-blown CTE.^22^

## IS HEADING THE MAIN CAUSE OF CTE IN RETIRED SOCCER PLAYERS?

The first impression is that heading could have been the cause of CTE in the reported cases. The idea that the heavier leather balls of the past could have been more dangerous than today's synthetic balls is probably unfounded, because synthetic balls can reach higher speeds. Further studies are needed to evaluate the risks of small but frequent concussions caused by heading. So far, studies have been inconclusive in implicating changes in the way soccer is played.^21^^,^ 23 ^,^ 24 There is limited evidence that heading in youth soccer players can cause concussion. The U.S. Youth Soccer recommendations are to teach heading after age 10 in controlled settings, and heading in games should be delayed until skill acquisition and physical maturity allow youth players to head correctly with confidence.^25^

## OTHER IMPACTS TO THE HEAD

Other head impacts in football may be more important than heading, particularly head to head contact.^26^ Traumas to the head, face and neck are among the most frequent in soccer. During the 2014 FIFA World Cup, the second-most-injured body part was the head, face and neck, accounting for 18% of all traumas, after 25% for the thigh.^27^

Many of these traumas can be avoided, particularly those of the elbow or other body parts against the head. Even head to head contact may be avoidable when a player is aware that they will not reach the ball with their head before the other player does.

In any event, after a concussion the player should be withdrawn from the game, a consensus statement that was not heeded even at the last FIFA World Cup.^28^

## A RECOMMENDATION

When a player makes contact with an opponent's leg during a challenge for the ball, they are penalized, sometimes severely with a yellow or red card. The punishment for causing an avoidable trauma to another player's head should be even more severe.

To conclude, there is now reasonable evidence for neurologists and Neurological Associations to recommend more severe punishment (red card and suspension for several matches) for players causing avoidable trauma to an opponent's head.
